# High risk of unsuccessful treatment outcome in migrant population with tuberculosis: Data from three Italian hospitals

**DOI:** 10.3389/fpubh.2022.1024474

**Published:** 2023-01-10

**Authors:** Francesco Di Gennaro, Sergio Cotugno, Massimo Fasano, Aurelia Ricciardi, Luigi Ronga, Rossana Lattanzio, Anna Grimaldi, Davide Fiore Bavaro, Marianna Ciarallo, Stefania Garzone, Giuseppina De Iaco, Giacomo Guido, Josè Ramon Fiore, Gaetano Brindicci, Carmen Rita Santoro, Salvatore Sica, Tiziana Loredana Iacovazzi, Teresa Antonia Santantonio, Annalisa Saracino

**Affiliations:** ^1^Clinic of Infectious Diseases, Department of Biomedical Sciences and Human Oncology, University of Bari “Aldo Moro,” Bari, Italy; ^2^Unità Operativa Complessa (UOC) Infectious Diseases Azienda Sanitaria Locale Bari (ASL BA), Bari, Italy; ^3^Microbiology and Virology Unit, University Hospital Policlinico, University of Bari, Bari, Italy; ^4^Chemical-Clinical and Microbiological Research Unit, ASL BA, Bari, Italy; ^5^Infectious Diseases Unit, Department of Clinical and Surgical Sciences, University of Foggia, Foggia, Italy

**Keywords:** migrant populations, high risk of treatment failure, vulnerables, lost-to-follow-up (LTFU), anti-tubercular treatment, tuberculosis

## Abstract

**Introduction:**

Tuberculosis (TB) remains an unresolved global health problem and vulnerable groups such as migrants remain the most affected with a higher risk of worse outcomes. The aim of this study was to evaluate clinical features, outcomes, and adverse events in migrant and native Italian patients admitted to three Italian hospitals in Southern Italy in order to assess differences and targeted strategies.

**Methods:**

We performed a retrospective study on TB patients admitted between January 1, 2013, and December 31, 2021, in three Apulia hospitals. Two logistic regression models were used, with the dependent variables being (I) unsuccessful treatment (died, loss to follow-up, and failed treatment) and (II) adverse events.

**Results:**

We enrolled 543 consecutive patients admitted at three Italian hospitals with a diagnosis of TB during the study period, of them 323 (59.5%) were migrants and 220 Italian patients. The treatment success rate in the migrant group was 44.9% (137/305), while in the non-migrant group was 97.1% (203/209). Independent factors of unsuccess treatment (death, failure or loss to follow up) were: migrant status (O.R. = 11.31; 95% CI 9.72–14.23), being male (O.R. = 4.63; 95% CI 2.16–6.10), homelessness (O.R. = 3.23; 95% CI 2.58–4.54), having a MDR (Multidrug-resistant) (O.R = 6.44; 95% CI 4.74–8.23), diagnostic delay (O.R. = 3.55; 95% CI 1.98–5.67), and length of hospitalization (O.R. = 3.43; 95% CI 1.88–5.87). While, age >65 ys (O.R. = 3.11; 95% CI 1.42–4.76), presence of extrapulmonary TB (O.R. = 1.51; 95% CI 1.31–2.18), monoresistance (O.R. = 1.45; 95% CI 1.25–3.14) and MDR pattern (O.R. = 2.44; 95% CI 1.74–5.03) resulted associated with adverse events.

**Conclusion:**

Migrant population is at high risk of unsuccessful treatment (death, loss to follow-up, and treatment failure). Policies targeted specifically to this group are needed to really impact and improve their health status and also to contain the TB burden.

## Introduction

Tuberculosis (TB) remains an unresolved global health problem. It is estimated that in 2020 around 10 million individuals became infected with *Mycobacterium tuberculosis* (*M. tb*) and 1.6 million died due to TB (1). Moreover, the impact of the COVID-19 pandemic on TB services is estimated to be dramatic, having destroyed the improvements achieved in the control of this disease over the last 10 years (1). Some authors defined TB as a disease of poverty and few diseases reflect and express social inequalities in their distributions and outcomes as TB (2). Vulnerable groups such as migrants, refugees, homeless, and people with low social status remain the most affected with a higher risk of loss to follow-up and worse outcomes. Furthermore, poverty is creating an ecosystem in which TB becomes more prevalent, deadlier, and harder to treat. Poverty and its related factors affect the risk of infection with *M. tb*, as well as the likelihood and severity of progression to active disease, the access to and quality of health care, and the ability to adhere to and complete the treatment (2).

Poverty, migrant status, and poor TB outcomes are strongly related (3, 4). In fact, many studies showed how poverty was related to a high diagnostic delay, and the worst health outcomes for communicable and non-communicable diseases (5, 6). In addition, migrants have key risk factors for TB and poor TB outcomes, such as poverty, poor, and dangerous working conditions, limited access to health-care services, and social exclusion, among other factors (7, 8). This makes migrant population a very vulnerable group.

Italy is a low endemic country for TB, with an incidence of 7.1 cases per 100,000 people, and about 60% of cases are attributed to immigrants from regions with a high rate of tuberculosis and a high prevalence of multidrug-resistant (MDR) or extensively drug-resistant TB (XDR), such as Africa, Asia and Eastern Europe (9, 10). In order to improve burden control and define the burden and transmission of TB in Italy, it is critical to investigate the patient characteristics in the most vulnerable groups, such as migrant, with a focus on the treatment success rates. Thus, we conducted a retrospective study evaluating clinical features, outcomes, and adverse events in migrant and native Italian patients admitted to three Italian hospitals in Southern Italy in order to assess differences and target strategies.

## Materials and methods

### Study design, study setting, and patients

According to WHO guidelines, enrolled patients were diagnosed with “active TB” on the basis of the following criteria: (I) a positive culture for *M. tb* from a respiratory sample (sputum or bronchoalveolar lavage) or other biological specimens; (II) a positive *M. tb* nucleic acid amplification test (NAAT) (GeneXpert), (III) evidence on histological examination (e.g., of a lymph node or other anatomic site) of caseous necrosis material with positivity using the Ziehl-Neelsen method; (IV) “clinical TB” when the diagnosis was based on clinical and radiologic criteria (after ruling out other illnesses), as well as on an adequate response to conventional anti-TB medication.

Patients were treated according to WHO TB guidelines (11). In particular, patients received directly observed therapy (DOT) during hospitalization. Discharged patients were followed monthly in outpatient care by qualified TB experts for the duration of therapy.

Laboratory tests were conducted monthly or as clinically necessary until the end of therapy. Potential adverse events of treatment and the need for timely patient reporting of any adverse events were discussed.

### Data collection

We performed a retrospective study on TB patients admitted between January 1, 2013, and December 31, 2021, in three Italian hospitals: (I) Clinic of Infectious Diseases, University of Bari, Bari, Italy, (II) Clinic of Infectious Diseases, University of Foggia, Foggia, Italy, (III) St Clinic of Infectious Diseases at Fallacara Hospital, Triggiano, Bari, Italy.

The primary data sources were patients' medical records. Admission and discharge/death dates, demographics, and clinical characteristics such as symptoms, tuberculosis diagnosis, *M. tb* drug resistance, TB site, treatment regimen, type of adverse events, and outcomes were collected and registered in an electronic database.

For the purpose of the analysis, due to the heterogeneity of the data from different centers and the low number of patients, treatment outcomes for TB patients' were grouped as follows: (I) *successful treatment*, as the sum of “cured” and “treatment completed” as for the WHO definitions (12), (II) *unsuccessful treatment*, comprising patients meeting the WHO definitions (12) of “treatment failed,” “died,” and “loss to follow-up.”

### Statistical analysis

No formal sample size was calculated *a priori* since the study included all patients admitted during the study period. Continuous data were expressed as the median and interquartile range (IQR), and categorical data as numbers and percentages. Chi-squared test or Fisher's exact test as appropriate was used to compare categorical variables, while *t*-test was used to compare continuous variables. We stratified our cohort into two groups: migrants and non-migrants in order to explore any differences between these two groups.

According to the International Organization for Migration (IOM), we defined “migrant” as any person who is moving or has moved across an international border or within a state away from his/her habitual place of residence, regardless of (I) the person's legal status; (II) whether the movement is voluntary or involuntary; (III) what the causes for the movement are; or (IV) what the length of the stay is United Nations (13) and Di Gennaro et al. (14).

As for treatment outcomes, patients whose treatment was still ongoing at the end of the data collection phase were excluded from the calculation of the analysis.

Two logistic regression models were used, with the dependent variables being (I) unsuccessful treatment (death, loss to follow-up, and failed treatment) and (II) adverse events, and the independent variables being each of the available parameters (univariate analysis).

In the univariate analysis, all covariates having a *p* < 0.10 were included in the model. The variance inflation factor (VIF) was used to examine multicollinearity among variables, with a value of two indicating that a covariate should be excluded. According to the prior criterion, however, no variable was eliminated.

The connection between covariates at the baseline (exposure) and unsuccessful treatment was measured using odds ratios (ORs) and adjusted odds ratios (Adj–ORs) with their 95 percent confidence intervals (CIs) (outcome). All two-tailed tests *p* < 0.05 were considered statistically significant. Statistical analysis was performed using STATA V.13.

## Results

Between January 1, 2013, and December 31, 2021, 543 consecutive patients admitted at three Italian hospitals with a diagnosis of TB, were included in the study. The sample was composed of 323 (59.5%) migrants (median age: 25.5 years) and 220 Italian patients (median age: 47.5 years). In addition, 380 patients (70%) were male, 113 (20.8%) were ≥65 years old, and 93 (17.1%) were homeless. Pulmonary TB was diagnosed in 340 (62.6%) patients, of whom 332 (61.1%) manifested respiratory symptoms. Furthermore, 385 (70.9%) were culture positive, 24 (4.4%) were HIV co-infected, 88 (16.2%) showed at least one drug resistance, and 31 (6.4%) had an MDR pattern.

**Figure 1:**
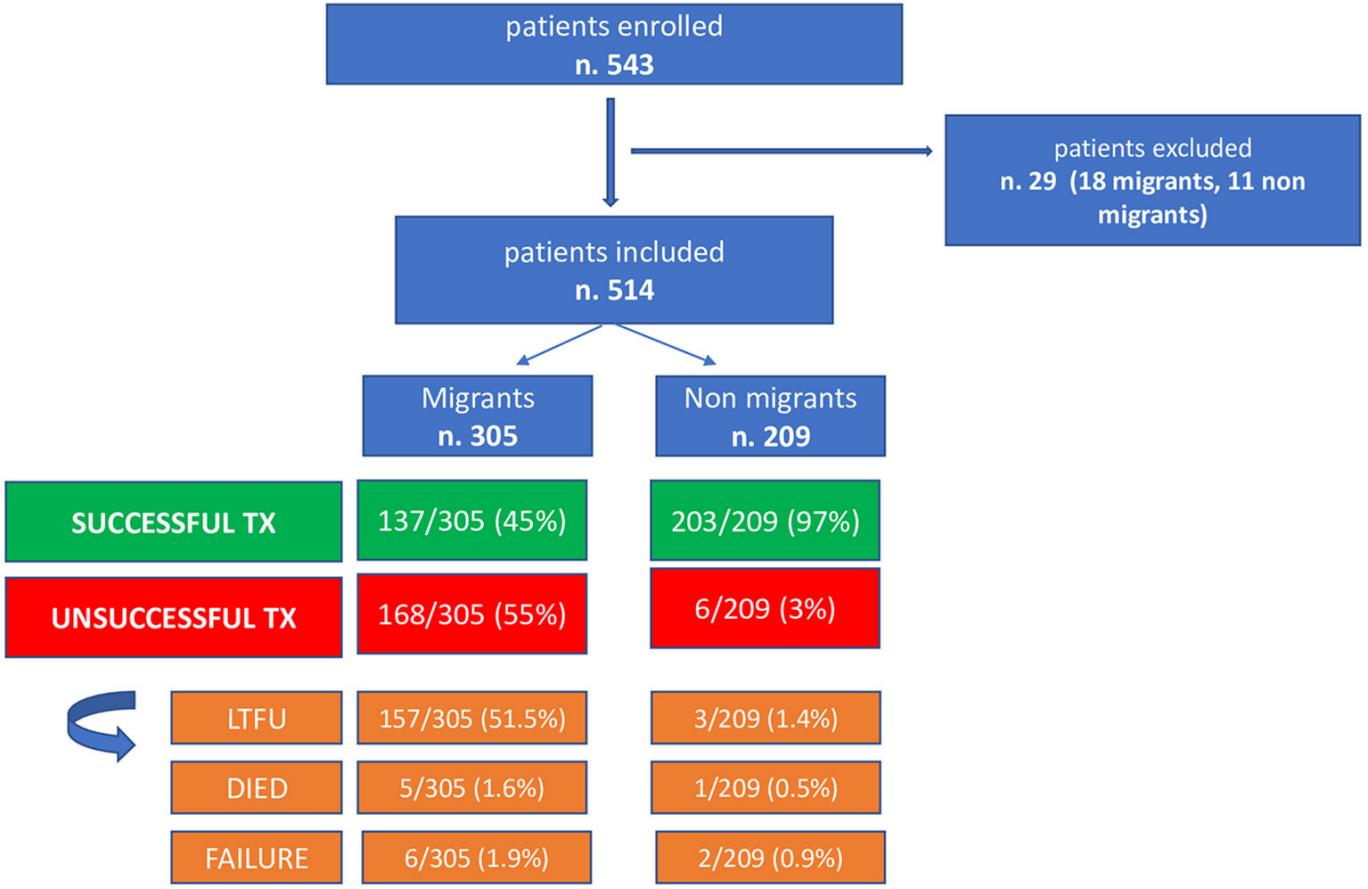
Patients outcome enrolled in the study.

At the time the data collection for the study finished, treatment was still ongoing in 5.3% (*n* = 29) of the sample, thus they were excluded from the analysis of outcomes. Thus, the treatment success rate in the migrant group was 44.9% (137/305), while in the non-migrant group it was 97.1% (203/209) (Figure 1).

Exploring the reasons for the treatment failure in the migrant group, we found that *n*. 157 (51.5%) were LTFU, 5 (1.6%) died and 6 (1.9%) were treatment failure.

Demographic, clinical, and therapeutic data of patients stratified by migrant status are reported in Table 1. Differences in the distribution of the variables between migrants and non-migrants were detected for male sex (*p* = 0.01), median age (*p* ≤ 0.001), age over 65 (*p* ≤ 0.001), homeless (*p* = 0.00), HIV positive (*p* ≤ 0.001), previous contact with a TB patient (*p* = 0.01), length of hospitalization (*p* = 0.01), diagnostic delay (*p* = 0.01) and unsuccessful treatment (*p* = 0.00). Furthermore, a significant difference was also observed in the drug resistance pattern (Table 1).

**Table 1:** Demographic, clinical, and therapeutic data of 543 patients stratified by migrant status.

		**Enrolled patients**			***p*-value**
		**Total** ***n*****. 543 (100%)**	**Migrant** ***n***. **323 (59.5%)**	**Non-migrant** ***n*****. 220 (40.5%)**	
Hospital	Bari	204 (37.5)	101 (49.5)	103 (50.5)	–
	Foggia	245 (45.1)	146 (59.6)	99 (40.4)	–
	Triggiano	94 (17.4)	76 (80.8)	18 (19.2)	–
Sex	M	379 (70)	247 (65)	132 (35)	0.01
	F	164 (20)	76 (46.3)	88 (33.7)	
Age years,					0.00
Median (IQR)		45.5 (18–92)	25.5 (18–75)	47.5 (18–92)	
Mean (SD)		39.8 (➅18)	27 (➅12)	43 (➅9)	
Age > 65 yrs *N* (%)		113 (20.8)	23 (20.3)	90 (79.7)	0.00
Homeless *N* (%)		93 (17.1)	92 (99)	1 (1)	0.00
HIV + status *N* (%)		24 (4.4)	21 (87.5)	3 (12.5)	0.00
Previous contact with TB patient *N* (%)		166 (21.4)	62 (37.3)	104 (62.7)	0.01
Length of hospital stay days, median (range)		44.5 (4–235)	43 (14–235)	24 (4–54)	0.01
Diagnostic delay, days					0.01
Median (range)		76 (5–500)	190 (30–500)	63 (5–170)	
Mean (SD)		102.8 (➅25)	139 (➅18)	39.3 (➅12)	
Type	Pulmonary TB	340 (62.6)	175 (51)	165 (49)	0.72
	Extrapulmonary TB	168 (31)	115 (68.4)	53 (31.6)	0.02
	Miliary TB	35 (6.4)	33 (99)	2 (1)	0.00
Respiratory symptoms		332 (61.1)	201 (60.5)	131 (39.5)	0.71
Type of diagnosis	Culture positive	385 (71)	201 (52.2)	179 (47.8)	0.06
	Radiological	109 (20)	83 (76.2)	26 (33.8)	0.03
	NAT	17 (3)	12 (70.5)	5 (29.5)	0.40
	Histological	32 (6)	22 (68.8)	10 (21.2)	0.32
	IGRA test	337 (62)	198 (58.7)	139 (41.3)	0.62
Initial therapeutic scheme, *n* (%)	R + H + E + Z	393 (72)	199 (50.6)	194 (49.4)	0.93
	Drug regimen without Z including amikacin	14 (2.6)	8 (57.1)	6 (42.9)	0.60
	Drug without Z regimen including fluoroquinolone	17 (3)	9 (53)	8 (47)	0.61
Resistance pattern	Monoresistance	88 (16)	77 (87.5)	11 (22.5)	0.01
	H	31 (5.7)	26 (83.8)	5 (16.2)	0.01
	R	46 (8.5)	41 (89.1)	5 (10.9)	0.01
	Z	11 (2)	10 (99)	1 (1)	0.05
	MDR	31 (6.4)	30 (99)	1 (1)	0.00
Adverse events and type, *n* (%)	Adverse events	136 (25)	46 (33.8)	91 (66.2)	0.01
	Hepatitis	85 (15.6)	30 (35.3)	55 (64.7)	0.02
	Neurological	13 (3)	7 (53.8)	6 (46.2)	0.81
	Itching/skin rash	18 (3.3)	3 (16.6)	15 (83.4)	0.01
	Cardiological	12 (2.2)	3 (25)	9 (75)	0.02
	Ocular damage/decrease in visual acuity	2 (0.4)	1 (50)	1 (50)	1.00
	Acute renal failure	6 (1.1)	2 (34)	4 (66)	0.80
	Therapeutic shift	65 (12)	25(38.5)	40 (61.5)	0.01
Outcomes *N* = 514 (305 Migrant, 209 Non-migrant)	Successful treatment	340 (66.1)	137 (45)	203 (97)	0.00
	Unsuccessful treatment	174 (33.9)	168 (55)	6 (3)	0.00
	Lost-to-follow up	160 (31.1)	157 (51.5)	3 (1.43)	0.00
	Dead	6 (1.1)	5 (1.6)	1 (0.47)	0.01
	Failure	8 (1.5)	6 (1.9)	2 (0.95)	0.01
Treatment ongoing		29 (5.3)	18 (62.5)	11 (37.5)	0.82

One hundred thirty-six (25.0%) patients experienced adverse events related to the TB drug regimen; of whom 85 patients (62.5%) showed hepatotoxicity, requiring a therapeutic shift in 65 patients (47.8%). All adverse events are reported in Table 1.

The univariate and multivariate analyses on treatment outcomes included the following variables: migrant status, age, gender, homelessness, presence of respiratory symptoms, diagnostic delay, length of hospitalization, drug resistance, treatment regimen, TB localization (lung or extrapulmonary), and TB culture positivity. Significant predictors of unsuccessful treatment are reported in Table 2. Independent factors of unsuccessful treatment (death, treatment failure or loss to follow up) were: migrant status (O.R. = 11.31; 95% CI 9.72–14.23), male gender (O.R. = 4.63; 95% CI 2.16–6.10), homelessness status (O.R. = 3.23; 95% CI 2.58–4.54), monoresistant TB (OR = 3.75; 95% CI 2.45–5.24), MDR TB (O.R = 6.44; 95% CI 4.74–8.23), diagnostic delay (O.R = 3.55; 95% CI 1.98-5.67), and length of hospitalization (O.R. = 3.43; 95% CI 1.88–5.87) (Table 2).

**Table 2:** Predictors of unsuccessful treatment for active pulmonary tuberculosis.

**Characteristics**	**Univariate analysis O.R**.	**Multivariate analysis Adj-O.R**.
Migrant status	6.62 (4.98–11.64)	11.31 (9.72–13.23)
Age > 65	0.72 (0.48–1.64)	0.61 (0.42–0.76)
Male	2.48 (1.16–3.90)	4.63 (2.16–6.10)
Homeless	1.51 (1.28–2.03)	3.23 (2.58–4.54)
Diagnostic delay	2.72 (2.08–3.01)	3.55 (1.98–5.67)
Length of hospitalization	2.15 (1.68–3.54)	3.43 (1.88–5.87)
Pulmonary TB	1.33 (0.28–1.63)	1.29 (0.88–2.14)
Extrapulmonary TB	1.34 (0.68–1.88)	1.81 (0.91–3.18)
HIV status	1.19 (0.59–1.39)	0.88 (0.55–0.98)
Respiratory symptoms	1.36 (0.85–1.92)	1.23 (0.90–1.90)
Culture positive	1.44 (0.38–1.78)	1.64 (0.58–1.81)
Monoresistance, *n*	1.35 (1.12–1.60)	3.75 (2.45–5.24)
MDR	3.91 (2.19–5.13)	6.44 (4.74–8.23)
R + H + E + Z	1.59 (0.68–2.21)	1.80 (0.83–3.21)
Drug regimen without Z including amikacin	0.59 (0.48–1.21)	–

R, Rifampicin; H, Isoniazid; E, Ethambutol; Z, Pyrazinamide; TB, Tuberculosis.

The multivariate logistic model on the adverse events considered the effects of migrant status, age >65, gender, homelessness, presence of respiratory symptoms, diagnostic delay and length of hospitalization, drug resistance, treatment regimen and TB culture positivity, TB localization (lung or extrapulmonary). In fact, age > 65 years (O.R. = 3.11; 95% CI 1.42–4.76), extrapulmonary TB (O.R. = 1.51; 95% CI 1.31–2.18), monoresistant TB (O.R. = 1.45; 95% CI 1.25–3.14) and MDR TB (O.R. = 2.44; 95% CI 1.74–5.03) resulted associated with adverse events in our population, as reported in Table 3.

**Table 3:** Predictors of adverse events for active pulmonary tuberculosis.

**Characteristics**	**Univariate analysis O.R**.	**Multivariate analysis Adj-O.R**.
Migrant status	1.42 (0.68–1.64)	1.31 (0.79–1.63)
Age > 65	1.72 (1.48–2.64)	3.11 (1.42–4.76)
Male	1.48 (0.66–1.80)	1.69 (0.96–2.20)
Homeless	0.91 (0.78–1.93)	1.37 (0.78–1.93)
Diagnostic delay	1.22 (0.64–1.79)	1.45 (0.73–1.87)
Length of hospitalization	1.15 (0.88–1.54)	1.83 (0.95–2.42)
Pulmonary TB	1.43 (0.48–1.63)	1.89 (0.88–2.14)
Extrapulmonary TB	1.74 (0.88–2.08)	1.51 (1.31–2.18)
HIV status	1.29 (0.69–1.69)	1.12 (0.65–1.76)
Respiratory symptoms	1.45 (0.85–1. 82)	1.23 (0.90–1.90)
Culture positive	1.44 (0.38–1.78)	1.64 (0.58–1.81)
Monoresistance, *n*	1.35 (1.12–1.60)	1.45 (1.25–3.14)
MDR	1.91 (1.19–2.53)	2.44 (1.74–5.03)
R + H + E + Z	1.39 (0.71–2.11)	1.65 (0.63–2.05)
Drug regimen without Z including amikacin	0.59 (0.48–1.21)	–

R, Rifampicin; H, Isoniazid; E, Ethambutol; Z, Pyrazinamide; TB, Tuberculosis.

Table 4 reports the top ten countries of migrants with TB. One hundred ninety-eight patients (61.3%) came from African countries, while 58 patients (18%) from East Europe and 67 patients (20.7%) from Asian countries.

**Table 4:** Top ten countries origin of migrant with tuberculosis.

**Continent**	**Country**	**Total,** **323 (100%)**
**Asian**		67 (20.7%)
	Bangladesh	24 (7.4%)
	Filippine	21 (6.5%)
	Others	22 (6.8%)
**African**		198 (61.3%)
	Nigeria	33 (10.2%)
	Ethiopia	29 (9%)
	Senegal	25 (7.8%)
	Somalia	16 (5%)
	Guinea	14 (4.3%)
	Ghana	14 (4.3%)
	Others African countries	67 (21%)
**East Europe**		58 (18%)
	Romania	28 (8.7%)
	Georgia	11 (3.4%)
	Others European countries	19 (6%)

**Figure 2:**
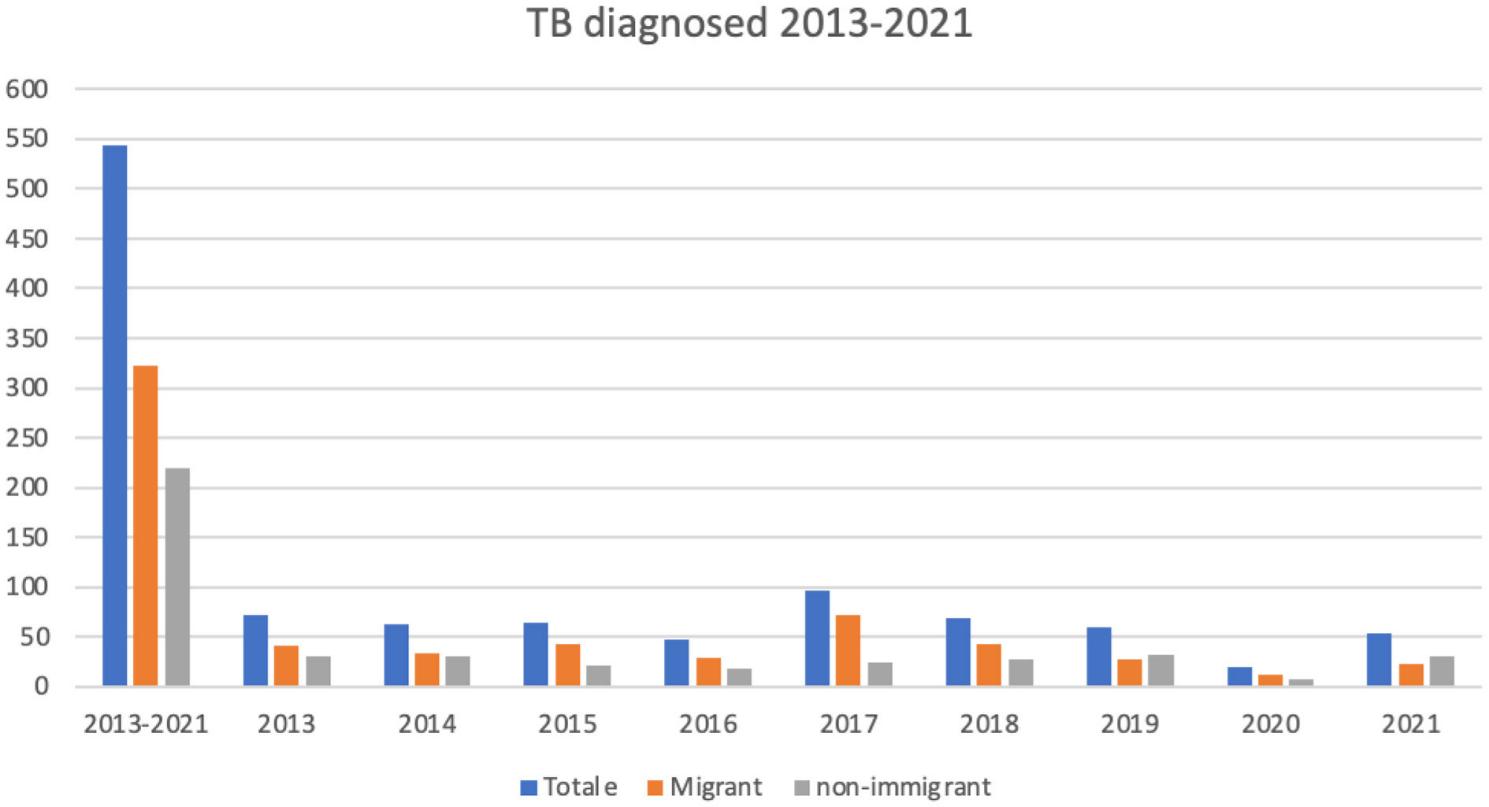
Temporal trend (2013–2021) of diagnosed TB in migrant and non-migrant population.

Figure 2 shows the temporal trend (2013–2021) of admitted TB patients by migrant status.

## Discussion

This study analyzes the data of TB patients admitted to three hospitals in Apulian geographical areas, where the migrants represent a consistent part of the general population; in Foggia, due to the presence of wide settlements related to the seasonal work in agriculture (15), and in Bari, as it is the regional capital. In accordance with the national data, in our study, the median age of migrants is definitely lower compared to the native group, and the male to female proportion is double among migrants compared to natives (16).

Our analysis shows that the risk of unsuccessful treatment in the migrant population is about 10 times higher compared to the non-migrant population. As many authors have already discussed, the migration status represents a negative health determinant for its multidimensional socio-economic characteristics: the unstable economic condition, the lack of a social network, lack of housing, and low income. All these factors contribute to an increase in the risk of poor health outcomes in TB management (17, 18). On the subject of this observation, we underline that homelessness increases the risk of unsuccessful treatment by more than three times. In our opinion, homelessness's negative effect could also be strongly related to diagnostic delay.

In fact, both homeless and migrant individuals often lack an appropriate living place, which delays their hospital discharge and prolongs the hospitalization stay. Moreover, these subjects often do not have access to the health system, which hinders the continuation of treatments (17–19).

As already acknowledged by other authors, the conversion of hospitalization into free housing services for homeless and poor people would represent a substantial and cost-effective public health policy (20). Furthermore, the development of a targeted diagnostic program for hard-to-reach populations proved to reduce both the diagnostic delay and the health costs (21).

Our data show that being male increases the unsuccessful treatment risk by four times. On the contrary, age over 65 years appears to be a protective factor for the unsuccessful treatment. The migrant population in our study is composed mainly of Africans and Asians engaged in agricultural work. This explains the major presence of young people and males in our migrant population. Although elderly patients are now considered a vulnerable target population, being older than 65 years in our study represents a protective factor against an unsuccessful treatment. This apparent contradiction can be explained by the younger median age and poverty of the migrant population, as other studies describe (10). On the other hand, the vulnerability of elderly people in our analysis is confirmed by the observation that being older than 65 years is a predictor of severe adverse events in TB by three times (22, 23). Thus, older age may be a risk factor when organic reasons play a major role (e.g., severe adverse events in the treatment of PTB), while it seems to be a protective factor when being older is an indicator of a better social condition.

In our study, multi-drug resistance and monoresistance increased the risk of unsuccessful treatment by more than 6 times and more than 4 times, respectively. This result can be explained by the major toxicity and complexity of drug-resistance treatment regimens, which led to a higher percentage of unsuccessful treatments (19, 24). On the other side, the high burden of DR-TB in countries of origin and the major frequency of previous undertreated TB episodes in the anamnesis of migrants, cause a more common occurrence of DR-TB among migrants than Italians (18, 25, 26).

Finally, we observed in our study that HIV status acts as a protective factor for the success of TB treatment, although this result contrasts with the general finding of poor outcomes in TB-HIV coinfected patients (27). We can explain this result from an integrated-care model perspective: being HIV positive often led the patient to a higher level of attention toward his health and the cooperation among pathology-specific units, which guarantees a more continuous prise-en-charge of the patient and a consequently more successful treatment.

We recognize some limitations of our study: the retrospective design of the study and the lack of some baseline clinical characteristics do not allow us to correctly identify the predictors of unsuccessful treatment. Nevertheless, the diagnostic delay among migrants may reduce the bias, as this condition correlates with a worse clinical status. Being a retrospective study, information about lost-to-follow-up (LTFU) patients could not be retrieved, and we admit that some patients, whom we consider LTFU, may have just continued the treatment at other centers.

## Conclusions

TB management cannot disregard the assessment and the intervention on social aspects, as the social determinants on their own prove to affect the outcomes more than disease-specific characteristics, such as drug resistance. Migration status is the best example of the context effect on treatment outcome. TB programs can be the solution to support immigrant people in all critical aspects of their existence to improve integration, self-determination, and access to services. This goal can be reached only if medical and non-medical interventions are equally considered at all levels of TB management (prevention, diagnosis, treatment, and follow-up). In addition, strengthening a national network for monitoring and tracking people with TB may be an effective strategy to maintain care and improve outcomes for this vulnerable population, even in the event of inter-regional mobility of TB patients. In addition, shorter regimens could be considered as regimens of choice for populations with a higher risk of loss to follow-up in order to reduce the treatment time while maintaining the same therapeutic efficacy. Moreover, consideration should be given to establishing at least one facility with nurse management per region or interregionally to allow frail TB patients (such as migrants and the homeless) to have access to accommodation, given that this could help them to remain adherent for the duration of antituberculosis treatment. Since migrant status and the related marginalized status often create a gap between health care providers in indigenous health care systems and migrants, integrating health welfare with health education programs could bridge the communication gap that alienates patients from TB care. For this reason, LTFU rates must be viewed as a consequence of both material and relational factors and should be approached as such. This could help to control the burden and reduce the occurrence of MDR or unsuccessful treatment. The solutions may be different from those proposed so far, but our proposal is to discuss them with institutions, associations, and other researchers in order to give TB patients a central role in the debate and find together the best possible health strategies. Endly, the immigrant population is at high risk of unsuccessful treatment (death, loss to follow-up, and failed treatment). Policies targeted specifically at this group are needed to really impact and improve their health status and also to contain the TB burden.

## Data availability statement

The original contributions presented in the study are included in the article/supplementary material, further inquiries can be directed to the corresponding author.

## Author contributions

FD, SC, AR, RL, GD, GB, CS, AS, DF, GG, LR, SG, TI, MF, SS, SAT, JF, MC, and AG contributed to conception and design of the study. FD, TI, MF, and SS organized the database. FD performed the statistical analysis. FD, SC, and AR wrote the manuscript. All authors contributed to manuscript revision, read, and approved the submitted version.
